# NPY and VGF Immunoreactivity Increased in the Arcuate Nucleus, but Decreased in the Nucleus of the Tractus Solitarius, of Type-II Diabetic Patients

**DOI:** 10.1371/journal.pone.0040070

**Published:** 2012-07-11

**Authors:** Nadia Saderi, Roberto Salgado-Delgado, Rafael Avendaño-Pradel, Maria del Carmen Basualdo, Gian-Luca Ferri, Laura Chávez-Macías, Juan E. Olvera Roblera, Carolina Escobar, Ruud M. Buijs

**Affiliations:** 1 Dept. de Biologia Celular y Fisiologia, Instituto de Investigaciones Biomedicas, UNAM, Mexico City, Mexico; 2 NEF-Lab, Dept. Cytomorphology, University of Cagliari, Monserrato (CA), Italy; 3 Facultad de Ciencias, UASLP, San Luis Potosì, Mexico; 4 Dept. de Anatomia, Facultad de Medicina, UNAM, Mexico City, Mexico; 5 Unidad de Neuropatología, Hospital General de Mexico, Mexico City, Mexico; Montreal Diabetes Research Center, Canada

## Abstract

Ample animal studies demonstrate that neuropeptides NPY and α-MSH expressed in Arcuate Nucleus and Nucleus of the Tractus Solitarius, modulate glucose homeostasis and food intake. In contrast is the absence of data validating these observations for human disease. Here we compare the post mortem immunoreactivity of the metabolic neuropeptides NPY, αMSH and VGF in the infundibular nucleus, and brainstem of 11 type-2 diabetic and 11 non-diabetic individuals. α-MSH, NPY and tyrosine hydroxylase in human brain are localized in the same areas as in rodent brain. The similar distribution of NPY, α-MSH and VGF indicated that these neurons in the human brain may share similar functionality as in the rodent brain. The number of NPY and VGF immuno positive cells was increased in the infundibular nucleus of diabetic subjects in comparison to non-diabetic controls. In contrast, NPY and VGF were down regulated in the Nucleus of the Tractus Solitarius of diabetic patients. These results suggest an activation of NPY producing neurons in the arcuate nucleus, which, according to animal experimental studies, is related to a catabolic state and might be the basis for increased hepatic glucose production in type-2 diabetes.

## Introduction

Animal studies have shown that the levels of circulating insulin and glucose are monitored in the Arcuate nucleus (ARC) of the hypothalamus and the caudal brainstem. The ARC is also referred to as Infundibular nucleus in the human brain. Changing concentrations of insulin and glucose modify the activity of several neuronal populations within these areas. These nutrient/hormone sensitive neurons project to pre-autonomic neurons located in the hypothalamus and brainstem and modulate the autonomic output to liver and pancreas. In both areas, neurons were identified that are either activated or inhibited by changes in glucose availability [1], [2], [3], [4]. At present, the ARC is mainly characterized by two antagonistic neuronal groups: the Neuropeptide Y (NPY) and pro-opiomelanocortin (POMC) expressing cells. In experimental animals, NPY neurons in the ARC are activated by negative metabolic challenges, such as fasting and hypoglycemia [5], [6] and NPY signaling is required to stimulate hepatic glucose production in response to a decrease in plasma glucose levels [7], [8]. POMC neurons, in contrast, are activated by positive energy conditions and satiety factors, and promote catabolism and energy expenditure. POMC is the precursor of several neuropeptides of which alpha-melanocyte stimulating hormone (α-MSH) is the cleavage product that mediates the effects on metabolism [9]. VGF is a neuropeptide precursor co-expressed in NPY and POMC neurons of the rat arcuate nucleus. The ablation of the VGF gene produces a hyper phagic and hyper metabolic phenotype, and results in increased NPY and diminished POMC expression; typical of prolonged fasting [10].

**Table 1:** Clinical pathological details of control individuals.

Matching nr	Sex	Age	Brain weight (g)	Cause of the death	Other clinical problems
1	M	55	–	Pneumonia	Multiple myeloma
2	M	58	1244	Acute renal failure	Hypertension Renal insufficiencyProstatic carcinoma
3	M	41	1397	Acute renal failure	Obesity Hypertension Coronal and aortic atherosclerosis Nephrosclerosis Hemorrhagic gastritis
4	M	59	1229	Septic shock from perforated gastric ulcersand peritonitis	Hepatic cirrhosis Pulmonary emphysema Coronal atherosclerosis
5	M	76	1329	Pneumonia	Nephrosclerosis Thrombosis at left femoral artery Prostatic hyperplasia
6	M	60	–	Gastric hemorrhage	Alcoholic liver cirrhosis Hypertension
7	F	51	–	Pneumonia	Hypertension Renal insufficiencyUnilateral nephrectomy
8	F	53	–	Metastasis from ovary cancer	Hysterectomia (2 years before)
9	F	41	1273	Hypovolemic shock from gastric hemorrhage	Non-Hodgkin lymphoma
10	F	54	–	Acute renal failure	
11	F	69	1050	Pulmonary congestion	Hepatic cirrhosis Nephrosclerosis Coronal and aortic atherosclerosis Ovary cancer

**Table 2:** Clinicopathological details of type-II diabetic individuals.

Matching nr.	Sex	Age	Brain weight (g)	Illness duration (years)	Cause of the death	Other clinical problems
1	M	72	–	10	Septic shock from perforated gastric ulcers	Liver insufficiency Chronic pneumonia from nicotinism Chronic alcohol abuse
2	M	41	1160	15	Acute renal failure	Hypertension Coronal and aortic atherosclerosis Nephrosclerosis Necrosis of the right feet
3	M	66	–	20	Coma	Hypertension Atherosclerosis Nephrosclerosis Gangrene of the left hand
4	M	57	–	22	Pneumonia	Hypertension Renal insufficiency
5	M	60	–	15	Cardiovascular failure post surgeryfor the amputation of the left leg	Hypertension Renal insufficiency Nephrosclerosis
6	M	65	1330	12	Pulmonarythrombo-embolism	Pulmonary tubercolosis Peritonitis Hypertension
7	F	72	–	–	Myocardial infarction	Aortic atherosclerosis Hypertension Pulmonary emphysema Perforated gastric ulcers Hysterectomy
8	F	50	–	18	Pneumonia	Congestive heart insufficiency Renal insufficiency Hepatic cirrhosis
9	F	48	1227	–	Myocardial infarction	Hypertension Coronal and aortic atherosclerosis Lymphoid spleen hyperplasia
10	F	45	1189	23	Septic shock after pyelonephritis	Renal insufficiency
11	F	45	1186	–	Respiratory failure	Thoracic lymphoma

Notwithstanding the wealth of data present in experimental animals nearly nothing is known about the response and changes of these peptide containing-neurons in the human brain. Except for a study that examined the changes of NPY neurons in the ARC in obese humans [11] no information is available about the possible changes in NPY, α-MSH and VGF neurons in the ARC and NTS in the diabetic condition. We hypothesized that if animal studies have a predictive value for what may happen in the human, the metabolic alterations caused by, or causing diabetic illness may modify the expression of the metabolic neuropeptides in the brain and thus indicate the disturbance of insulin and glucose detection in the brain of the diabetic. Hereto, we investigated the expression of NPY, α-MSH and VGF in the post mortem brain of type-2 diabetic individuals which was compared with their expression in brains obtained from age and sex matched non-diabetic patients. Moreover, we investigated the co-expression of VGF in human NPY and POMC neurons. Finally, because noradrenalin (NA) is proposed to play an important role in glucose homeostasis [3], we verified the relationship of catecholaminergic neurons with NPY neurons in the brainstem of diabetic and non-diabetic individuals.

**Figure 1:**
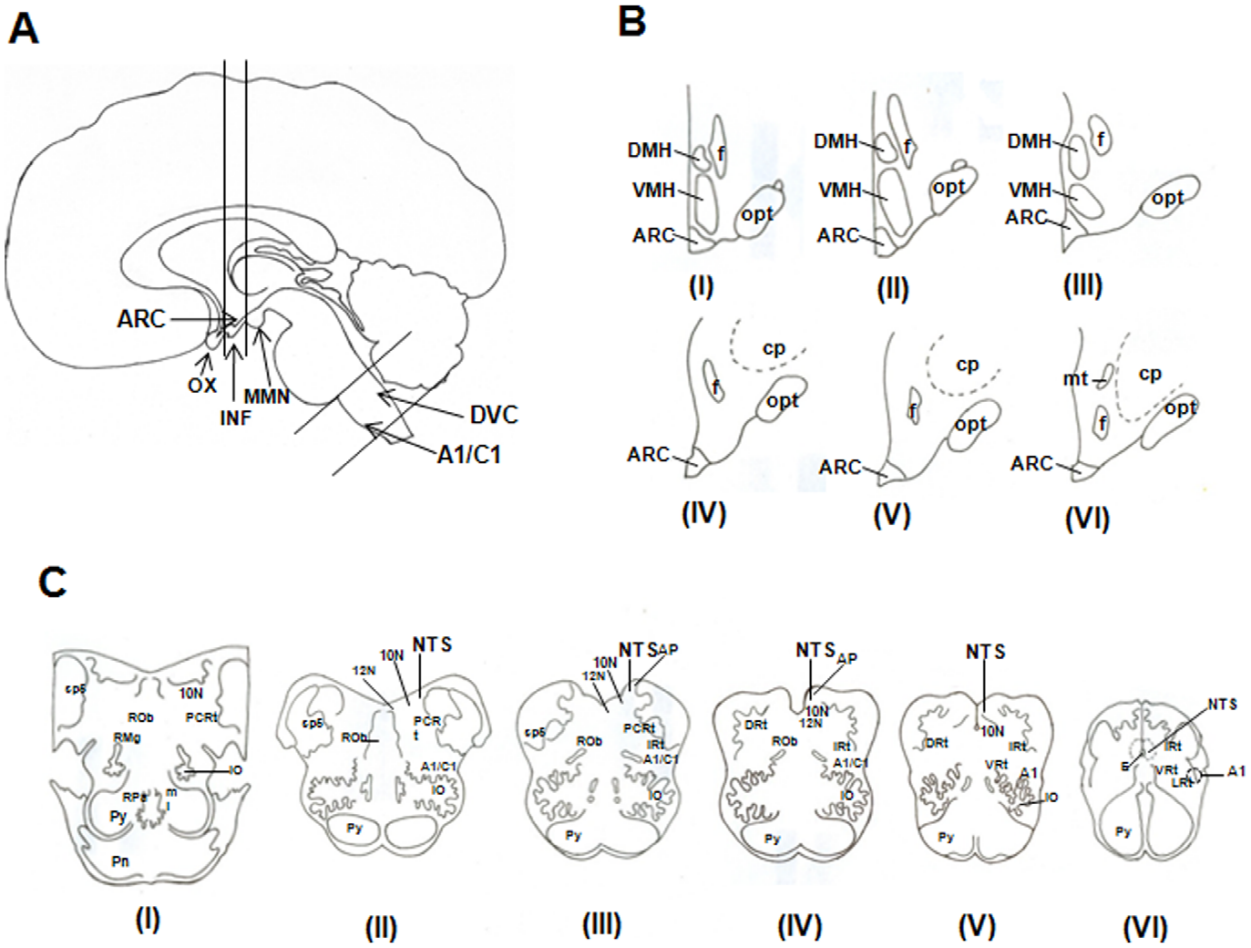
Schematic illustration of the human brain. a) sagittal view: areas included in the black lines were analyzed; b) semi-coronal illustration of the hypothalamus; c) coronal illustration of the medulla oblongata, all displayed in rostrocaudal order as indicated by the levels from I to VI (from 14,15,16). The stained area of the ARC was identified by: 1) the third ventricle at the midline; 2) the external layer of the median eminence and the ventral hypothalamic border; 3) the ventromedial hypothalamus clearly visible as having no NPY fibers. In the brainstem, the NTS region was delimited by: 1) dorsomedially, the fourth ventricle; 2) ventrolaterally, by the vagus nerve; 3) non-immuno positive areas in the other bordering areas. Abbreviations: 10N  =  Dorsal motor nucleus of the Vagus nerve; 12N  =  Hypoglossal nucleus; A1/C1 =  Noradrenergic/adrenergic cell groups; AP  =  area postrema; ARC  =  Arcuate nucleus; cp  =  Cerebral peduncle; DMH  =  Dorsomedial hypothalamic nucleus; DRt  =  Dorsal reticular nucleus; DVC  =  Dorso-vagal complex; E  =  Sub-ependymal layer; f  =  fornix; Inf  =  Infundibulum; IO  =  Inferior Olive; IRt  =  Intermediate reticular nucleus; LRt  =  Lateral reticular nucleus; α-MSH  =  Melanocyte Stimulating Hormone α; MMN  =  Mammillary nucleus;ml  =  Medial lemniscus; mlf  =  Medial longitudinal fasciculus; mt  =  Mammillo-thalamic tract; NTS  =  Nucleus of the Tractus Solitarius; NPY  =  Neuropeptide Y; opt  =  Optic tract; och  =  Optic chiasm; PCRt  =  Parvocellular reticular nucleus; pn  =  Pontine nucleus; py  =  Pyramidal tract; RMg  =  raphe magnus nucleus; ROb  =  Raphe obscurus nucleus; RPa  =  Raphe pallidus nucleus; sp5 =  Spino-thalamic tract; TH  = Tyrosine Hydroxylase; VMH  =  Ventromedial hypothalamic nucleus; VRt  =  Ventral reticular nucleus.

The present results show that, especially where it concerns NPY neurons in the ARC, a significant increase in the number of neurons expressing NPY can be found in the postmortem human diabetic brain. These changes, when extrapolated to experimental animal studies, suggest that the human ARC in the diabetic state is reflecting a catabolic state and is possibly unable to sense high circulating glucose levels. In addition the results show that the same neuronal phenotype in the NTS area responds quite differently to the diseased diabetic state.

## Materials and Methods

### Human Brains

For this study, 22 human brains were examined, 11 brains from patients (age mean ± SEM: 60.1±4.3, 6 males; 52±5.1, 5 females) who died of complications of type II-diabetes (DBT group) and 11 brains of non-diabetic individuals (52.2±4.3, 6 males; 54.2±5.7, 5 females), who died of conditions not related with a chronic metabolic impairment, considered as controls (CTR group) (Table 1,2). Diabetic and control cases were age and sex matched, post mortem time due to hospital practice was always between 8 and 12 hours and brains of controls and diabetics were always stained in pairs to avoid staining intensity differences due to procedure.

**Figure 2:**
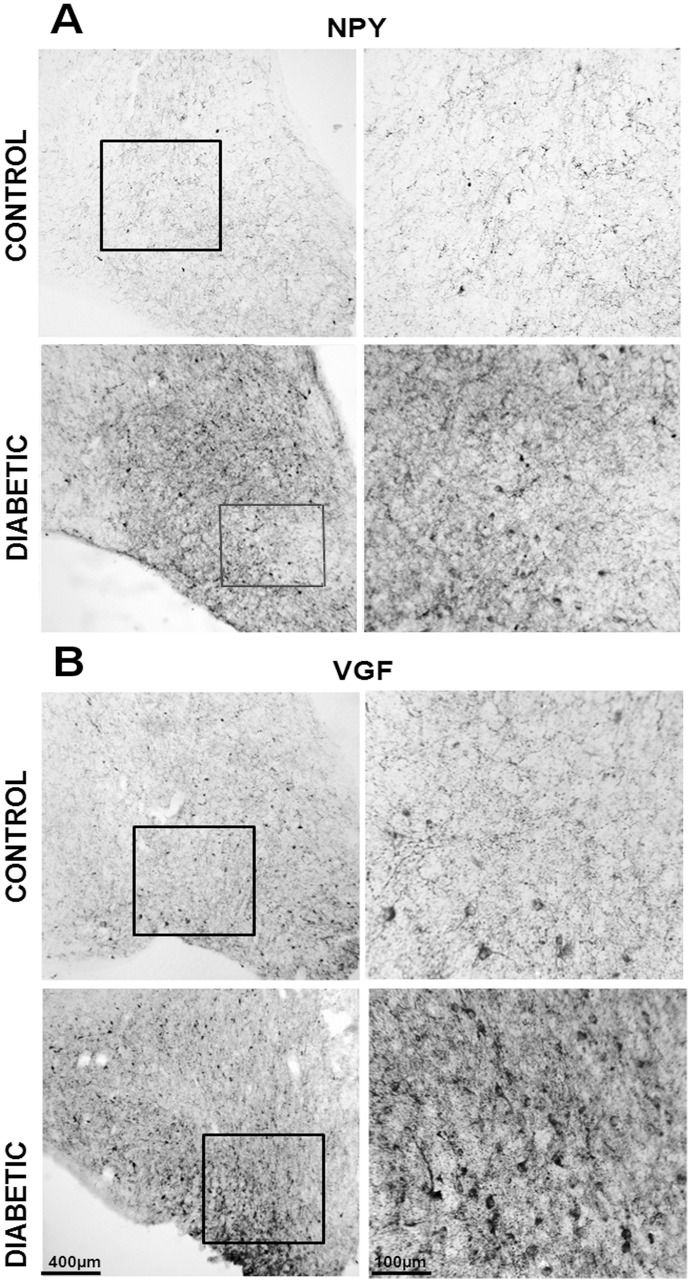
NPY and VGF immunoreactivity is up regulated in the diabetic ARC. Panel A demonstrates NPY and panel B VGF immunoreactivity in the ARC of control and diabetic patients, at level II and III respectively. Boxed areas are reproduced at higher magnification in the right columns. Scale bar  = 400 µm in the left columns;  = 100 µm in the right columns.

**Figure 3:**
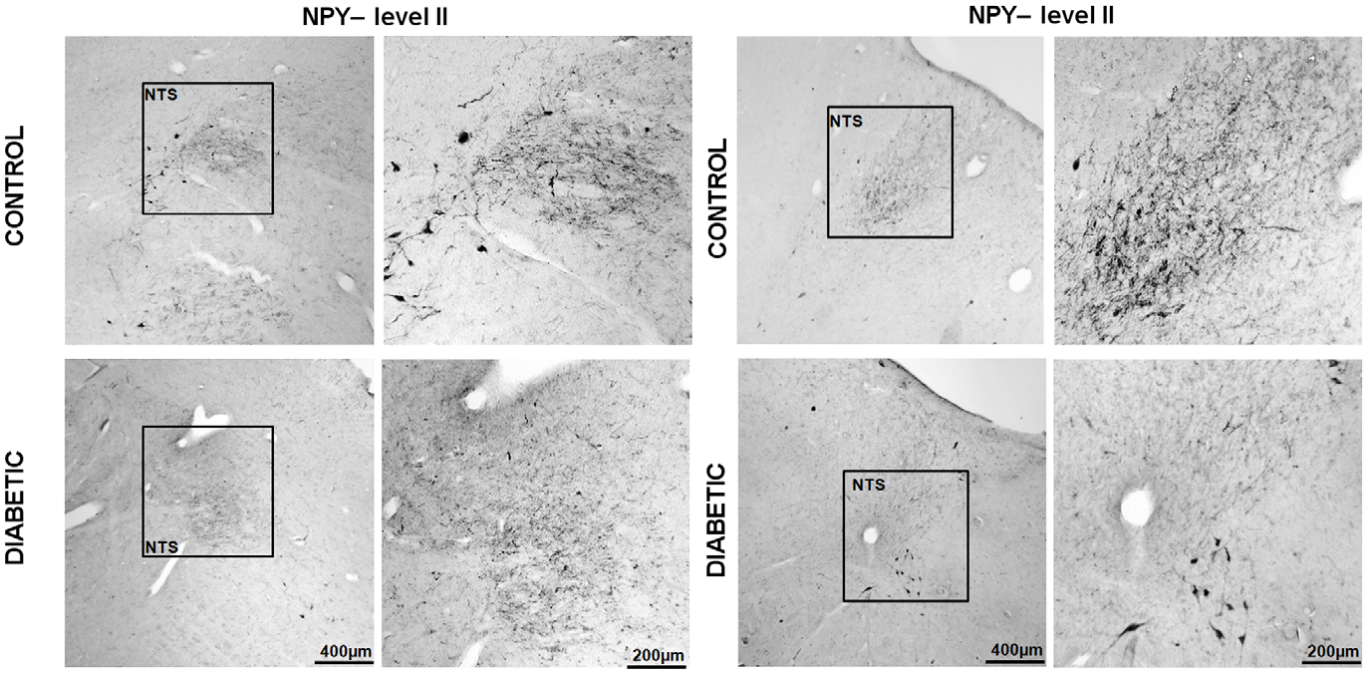
The diabetic NTS shows a lower NPY immunoreactivity in comparison to the control NTS. Panels A and B represent the levels II and III of the brainstem, respectively. Boxed areas are reproduced at higher magnification in the correspondent right columns of each panel. Note that these results are in contrast to what has been observed in the ARC. Scale bar = 500 µm in the left columns;  = 200 µm in the right columns.

**Figure 4:**
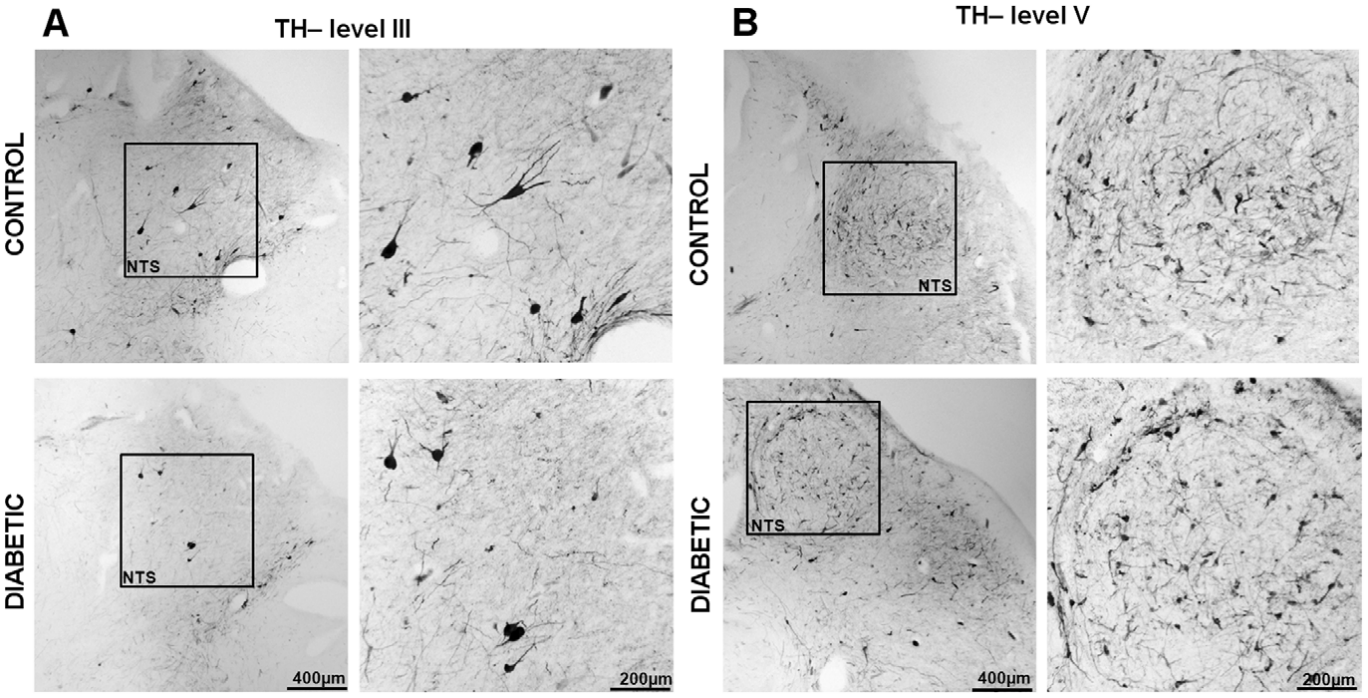
TH immunoreactivity in the diabetic NTS shows a tendency to decrease, but it does not differ significantly from the control. The series of pictures represents the levels III and V. Boxed areas are reproduced in the right columns at higher magnification. Note that big-sized TH- cells are present at higher levels of the NTS and the surrounding area, whereas smaller TH-positive cells are scattered in the whole area of the most caudal NTS. Scale bar  = 400 µ in the column A;  = 150 µm in the column B.

**Figure 5:**
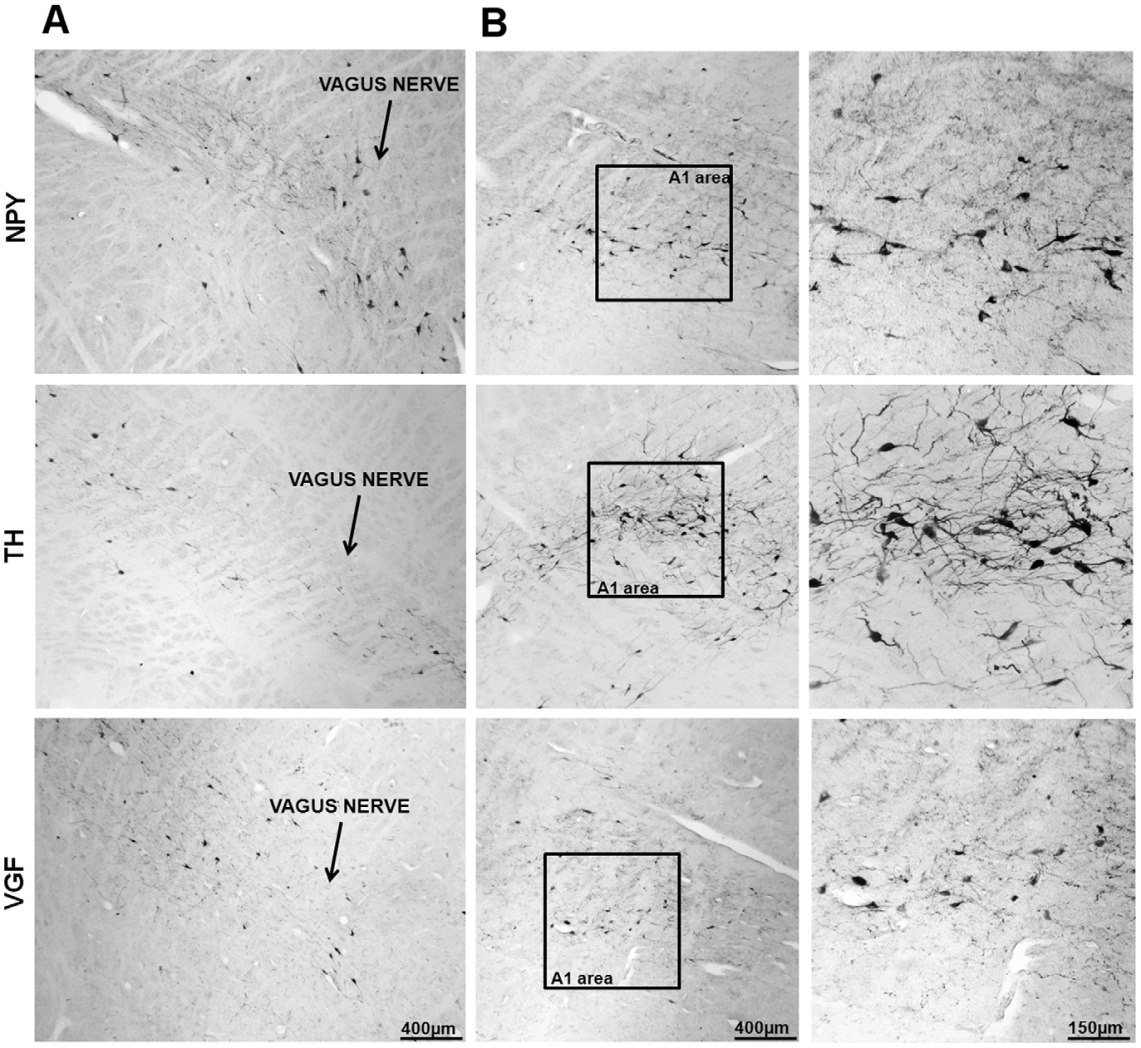
NPY, TH and VGF immuno stained neurons and fibers are observable along the pathway of the Vagus nerve and the A1/C1-area in the human medulla oblongata. Panel A: Vagus nerve; scale bar = 400 µm. Panel B: A-1 area, scale bar  = 400 µm; Panel B’: higher magnification of the boxed areas in the left column of the panel B; scale bar  = 150 µm.

**Figure 6:**
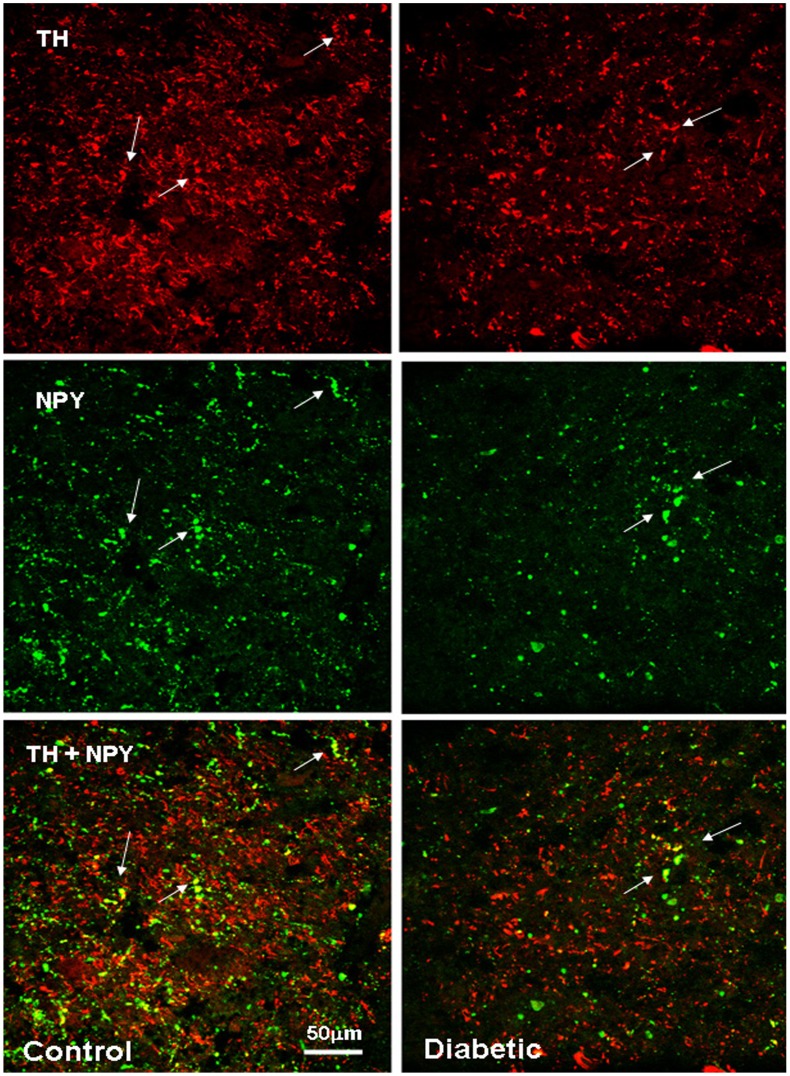
NPY and TH co-localizes in the human control and diabetic NTS. Confocal image of the NTS at level V: NPY and TH are visualized in green-Cy2 and red-Cy3, respectively. The white arrows indicate yellow co-localizing cellular elements. The co-localization of NPY and TH in the dorsovagal area was already reported in both rodent and human studies. Scale bar  = 50 µm.

**Figure 7:**
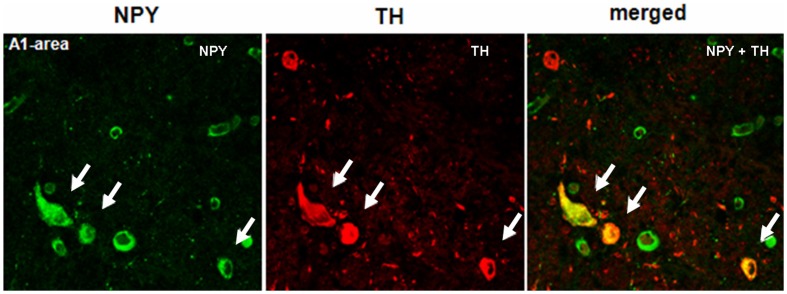
NPY and TH co-localize in some neurons in the human A1 area. Confocal image of the A1 area level IV: NPY and TH are visualized in green-Cy2 and red-Cy3, respectively. The white arrows indicate yellow co-localizing cell bodies. The main part of TH neurons within the catecholaminergic areas of the ventrolateral medulla is glucosensitive, and NPY expression in this A1/C1 cellular cluster is essential for glucoprivation-induced food intake in rats [3]. Scale bar  = 50 µM.

**Figure 8:**
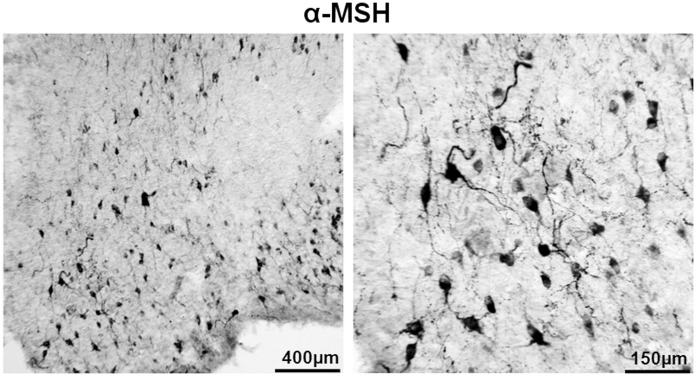
α-MSH big-sized neurons are present in the medio lateral portion of the ARC. Remarkably, α-MSH positive neurons are detectable also in the VMH and in a ventral area lateral to the ARC. Boxed area is reproduced in the right column. Scale bar  = 500 µm and 130 µm respectively.

Brains were collected by a team of neuropathologists at the Hospital General de Mexico in Mexico City from January 2004 to 2008. The protocol was revised and approved by the Ethical Committees of the Hospital General de Mexico and the Faculty of Medicine at Universidad Nacional Autonoma de Mexico, in according with the Mexican law Reglamento de la Ley General de Salud, Diario Oficial de la Federacion: D.O.F. 20-II-1985; D.O.F. 26-XI-1987; D.O.F. 06-1-1987. During hospitalization, Patients subscribed a written informed consent for the post-mortem utilization of organs, tissues and cells for Investigation at University, as it is required by the Mexican statute (Ley General de Salud, Titulo Decimo Quarto, Capitulo II, Articulo 321, Articulo 322). We should mention that most of the brains in the present study were obtained of patients of humble social origin and consequently the medication received was very limited or nil. Patients were admitted to the public hospital when their health was already seriously compromised and moreover most did not receive any long-term drug treatment for their pathologies and their complications. With the exception of three diabetic patients, from whom we don’t know how long they suffered type-2 diabetes, the rest of the individuals were diagnosed and affected by the diabetic disease for at least 10 years. The causes of death of the persons included in both the diabetic and control groups are mostly acute renal failure and severe infections, with some cases of respiratory insufficiency, gastrointestinal hemorrhages and myocardial infarction. Diabetic and control individuals were affected by similar complications elicited by a series of chronic pathologies, such as cardiovascular, renal and hepatic dysfunctions, sometimes associated with pneumonia or cancer.

**Figure 9:**
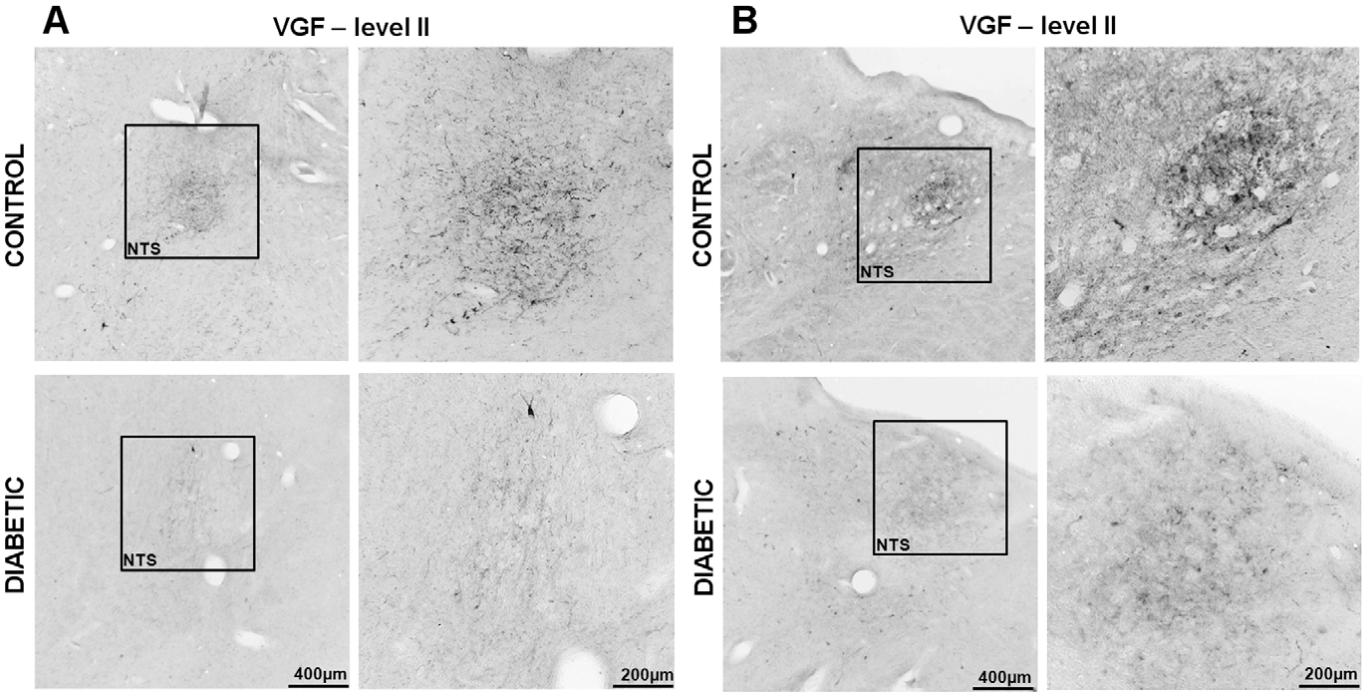
VGF immunoreactivity in the diabetic and control NTS. Panels A and B represent the levels II and III of the brainstem, respectively. Boxed areas are reproduced at higher magnification in the correspondent right columns of each panel. Note that these results are similar for NPY in both the observed areas (see figure 3). Scale bar = 500 µ in the left columns;  = 200 µm in the right columns.

### Tissue Processing and Immunohistochemistry

Brains were removed during routine autopsy always within 8–12 hours after the death. Brains were fixed by immersion in phosphate buffer (0.01 M, ph7.2) 4% paraformaldehyde (Sigma-Aldrich Corp., St. Luis, MO, USA) for two weeks. Dissected hypothalami and brainstems were stored in 4% paraformaldehyde for additional 30 days and then transferred to 30% sucrose- 0.04% NaN_3_ in Phosphate Buffer Saline (PBS, 0.01 M, ph7.6) at 4°C until sectioning. At −20°C the medio basal hypothalamus and the caudal brainstem were cut in slices of 50 µm such to obtain alternate sections of the ARC and the NTS. Sections were collected in 6 series and then stored in 30% sucrose- 0.04% NaN_3_ in PBS for immunohistochemical procedures.

**Figure 10:**
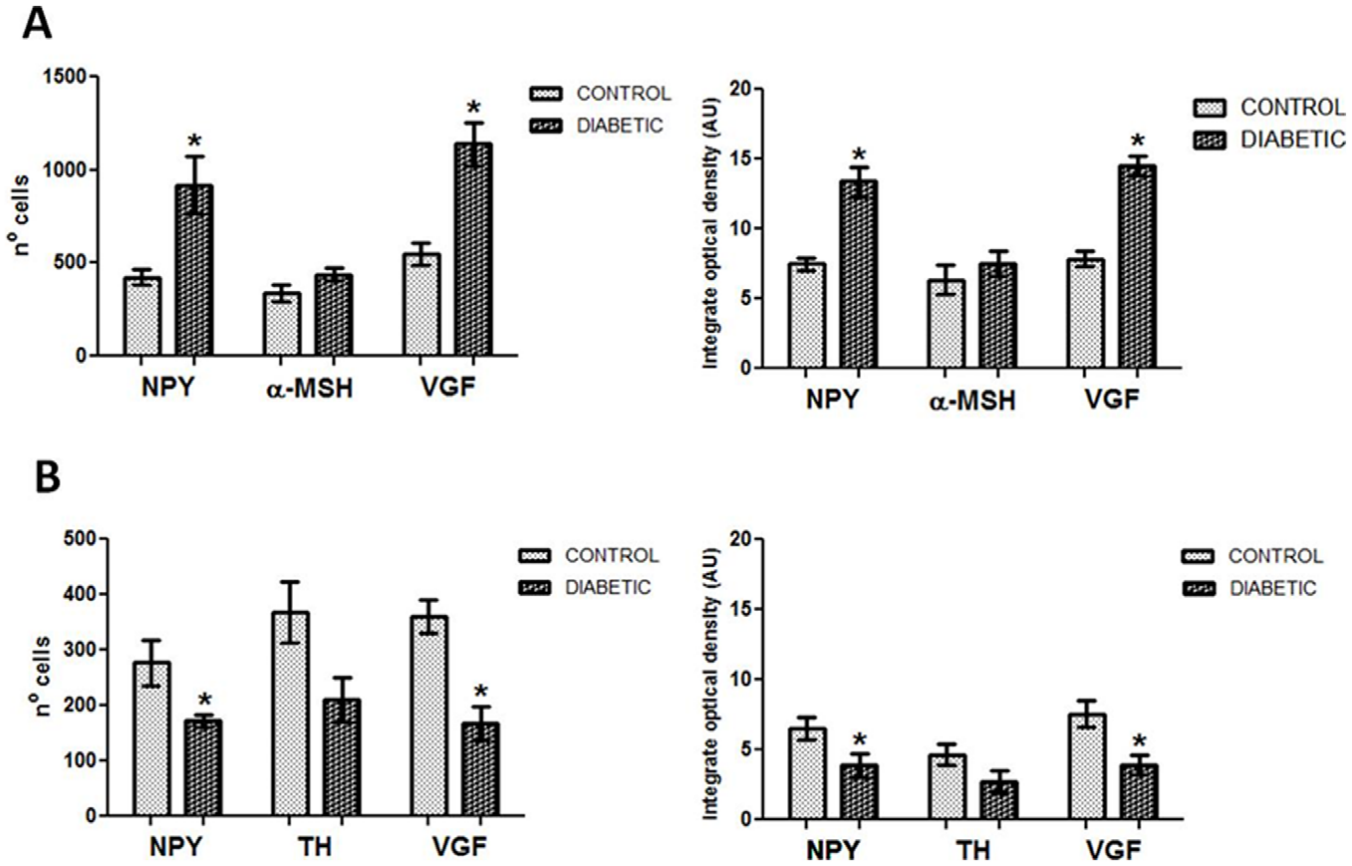
Quantification of the immunohistochemical staining. ARC NPY and VGF increases in the diabetic ARC, but decreases in the diabetic NTS. Panel A: the number of cells (right) containing NPY and VGF and the optical density (left) are higher in the ARC of diabetic individual, whereas α-MSH does not show any significant difference. Panel B: NPY and VGF positive cells (right) and their optical density (left) are reduced in the diabetic NTS. Note that also TH quantity shows a clear tendency to decrease, although the difference between control and diabetic groups is not statistically significant.

**Figure 11:**
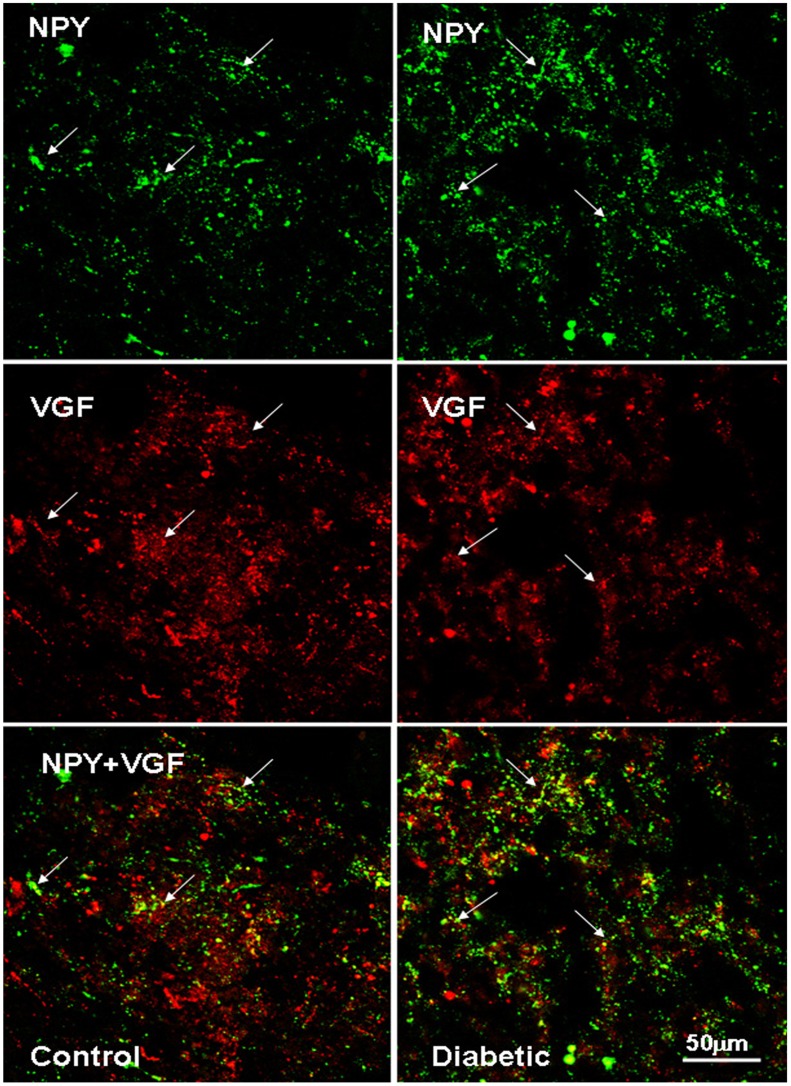
NPY and VGF co-localizes in some neuronal structures in the human ARC of control and diabetic subjects. Confocal laser scanning image of the ventromedial ARC: NPY and VGF are visualized in green-Cy2 and red-Cy3, respectively. The white arrows indicate yellow co-localizing cellular elements. Scale bar  = 50 µm.

**Figure 12:**
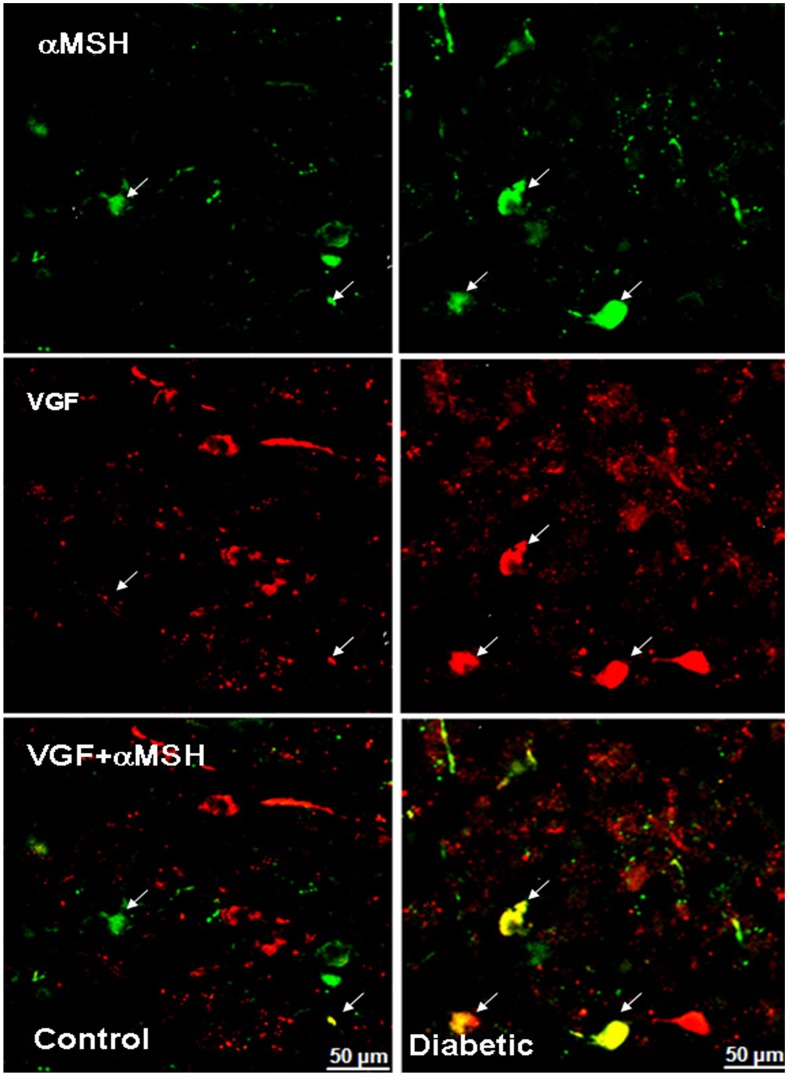
α-MSH and VGF co-localizes in some neuronal structures in human non-diabetic and diabetic ARC. Confocal image of the ventrolateral ARC: α-MSH and VGF are visualized in green-Cy2 and red-Cy3, respectively. The white arrows indicate yellow co-localizing cellular elements. Note that co-localization is appreciably increased in the diabetic ARC. Scale bar  = 50 µm.

**Figure 13:**
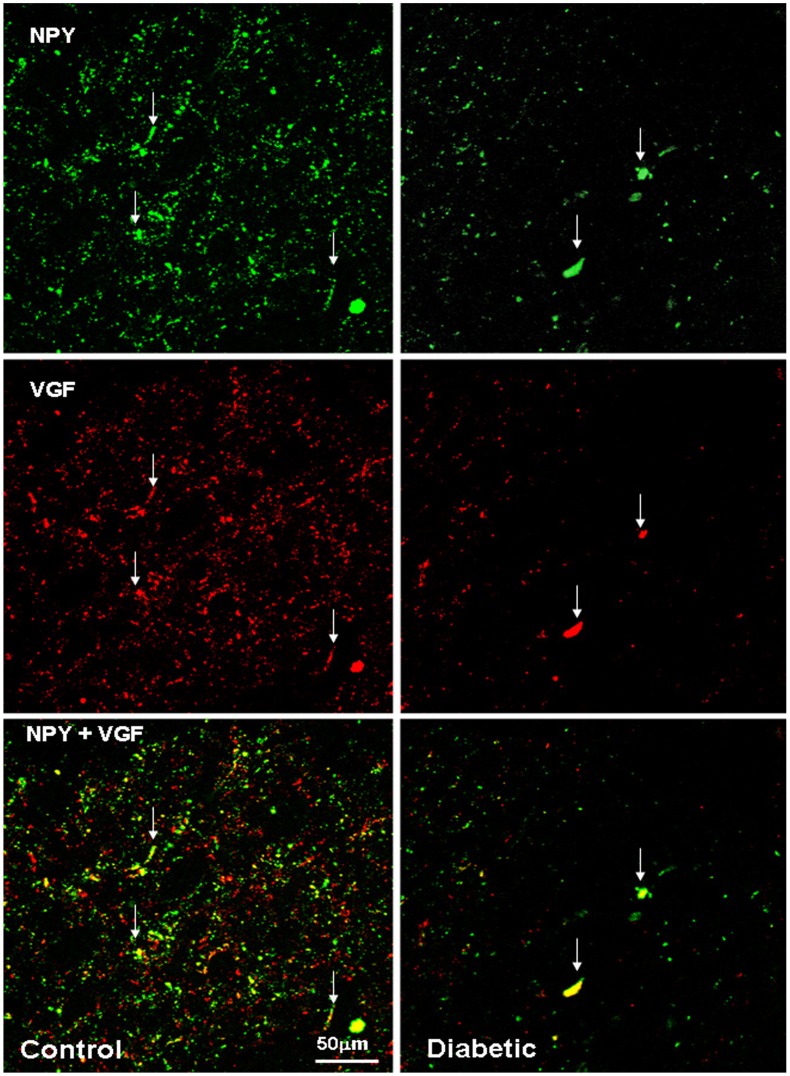
NPY and VGF co-localizes in some neuronal structures in the human NTS of control and diabetic subjects. Confocal image of the NTS at level III: NPY and VGF are visualized in green-Cy2 and red-Cy3, respectively. The white arrows indicate yellow co-localizing cellular elements. Note that diabetic NTS shows the decrease in both NPY and VGF staining already observed with DAB-peroxyde staining e.g. in figure 2. Scale bar  = 50 µm.

For each antibody, one series of 10 sections was used for immunohistochemical analysis, each section 300 µm apart, thus covering an area of 3 mm of the hypothalamus including the ARC or the dorsal vagus complex (Figure 1). Sections of controls and diabetic brains were always incubated together in order to avoid differences in staining intensities. Sections were incubated with either NPY, α-MSH or VGF antibodies. For this, a rabbit anti-NPY 1∶4000 (see for details on antibody specificity [12]) and a sheep anti- α-MSH, 1∶5000 (Chemicon International, Millipore Corporation, Billerica, MA, USA) were used. A rabbit antibody was raised against the human VGF C-terminal sequence VGF_609-615_, 1∶10000. [13]. A series of sections from the brainstem was incubated also with a Tyrosine Hydroxylase (TH), 1∶4000 (Millipore Corporation, Billerica, MA, USA) antibody. Dilution series of all antibodies were made and the optimal dilution was selected in such a way that never a saturation of the staining was obtained.

Antibodies were diluted 0.01 M Tris Buffered Saline (TBS), added with Triton X-100 0.5% (Sigma-Aldrich Corp., St. Luis, MO, USA) and gelatin 0.25% (Merck KGaA, Darmstadt, Germany), at room temperature for 1 h and then transferred at 4°C for 48 h. After rinsing, sections were incubated in biotinylated donkey-second antibodies,1∶400 (Jackson Immuno research, West grove, PO, USA) for 1.5 h, and then after extensive rinsing in PBS in avidin-biotin complex, 1∶500 (Vector Elite, Burlingame, CA, USA) for further 1.5 h. The final reaction was performed with a solution of 3,3′-diaminobenzidine 0.25% and H_2_O_2_ 0.01% (both Sigma-Aldrich Corp., St. Luis, MO, USA) in TBS, for 10′. Sections were mounted on gelatinized slides, dried and cover slipped with Entellan embedding agent (Merck KGaA, Darmstadt, Germany).

### Staining Quantification

To quantify the immunoreactivity, 10 sections of the ARC and 10 sections of the NTS were photographed at 40X and 63X magnification respectively and analyzed unilaterally for each neuropeptide. Digital pictures were taken using an Axioplan (Zeiss, Jena, Germany) microscope equipped with a digital color photo camera (Olympus DP25, Olympus, Japan). In order of identify the nuclei of interest the several bibliographic sources [14], [15], [16] were consulted and the areas delineated accordingly (Figure 1). The total optical density in cells and fibers as well as the total number of positive cells for each neuropeptide were counted with the program ImageJ. Data are represented as mean ± standard error of the mean (S.E.M.), analyzed with a one-way ANOVA analysis results were considered statistically different when they deviated from each other with a p<0.05.

### Fluorescent Immunohistochemistry

To investigate the co-localization of VGF with NPY and α-MSH, sections of the ARC and the NTS from at least three controls and three form the diabetic group were incubated for 48 h with rabbit anti-VGF antibody with either sheep anti-NPY or sheep anti-α-MSH (from Santa Cruz Biotechnology, Santa Cruz, CA, USA and Chemicon International, Millipore Corporation, Billerica, MA, USA, respectively). Sections from the brainstem of both groups were also incubated with rabbit anti-TH and sheep anti-NPY. Sections were then incubated with a mix of donkey anti-rabbit and donkey anti-sheep antibodies conjugated with fluorescent dyes (Cy3™- anc Cy2™-conjugated, respectively), all provided by Jackson Immunoresearch, West Grove, PO, USA. At the end of the reaction, sections were processed with 10% solution of Sudan Black (Sigma-Aldrich Corp., St. Luis, MO, USA) in ethanol, for 10′, to prevent autofluorescence. Finally, sections were mounted on gelatinized slides, cover slipped with 30% glycerol in PBS and analyzed with the LSM 5 Pascal confocal microscope (Zeiss, Jena, Germany).

## Results

### Ante Mortem Data

Unfortunately little or no ante mortem data such as glucose levels were available; for a part because 6 controls and one diabetic died from cardiovascular problems before they could reach the hospital. At the other hand blood glucose levels at admission were of special interest because they were less influenced by medication. In the controls glucose levels were in the range of 76–145 mg/dl with a mean of 100 mg/dl. The diabetics showed much more variation 72–1058 mg/dl with a mean of 228.1 mg/dl of which the low values were obtained after treatment with oral hypoglycemic drugs before hospitalization. Because of the missing values no statistical analysis could be done. Unfortunately also no data on body weight were available since such measurement is not routinely performed during hospitalization in this hospital.

### NPY, α-MSH and VGF in ARC and NTS of the Human Brain

The expression of NPY and POMC mRNAs, as well as the presence of NPY, α-MSH and TH peptides, in the human brain has been extensively described, and our results essentially concur with these descriptions [11], [17], [18], [19]. NPY cellular elements can be encountered throughout the whole diencephalon, but the ARC is one of the areas with the highest number of NPY-expressing neurons. In the ARC of both diabetic and non-diabetic patients, NPY positive neurons were scattered in the whole area of the nucleus (Figure 2-A). The distribution of NPY and TH neurons in the medulla oblongata also corresponds to what previously has been reported for the human brain [14], [23], [8], [20], [21]. In the brainstem, the majority of NPY and TH immuno reactive cells are found in the NTS and the ventro lateral medulla, whereby NPY is often co-expressed in catecholaminergic neurons (Figures 3, 4, 5, 6, and 7). In the hypothalamus, POMC expression has been reported to be restricted to the medio-lateral ARC [22], [23], [24]. However, our results indicate that α-MSH neurons, in addition to the ARC, are also present in the human ventromedial hypothalamus and in the adjacent ventro lateral area (Figure 8).

α-MSH, immunoreactivity was hardly detectable in the NTS of both diabetic and control groups. Indeed, no references about POMC expression in human brainstem are available in literature.

In humans, VGF mRNA has been observed in neural and endocrine cells [25]. We observed VGF-containing neurons in both ARC and NTS, as well as in the catecholaminergic areas of the brainstem (Figure 2-B, 5, 9). In our present study following the NPY, α-MSH and VGF immunohistochemical procedure, we have never seen any indication for a staining in glial cells, and as is also clear from the figures, only cells clearly identifiable as neurons were stained.

### NPY and VGF Show Higher Immunoreactivity in the ARC of type-2 Diabetic Subjects

The ARC of diabetic patients displayed a significant increase in the number and intensity of immuno reactive NPY cells as well as in the total NPY optical density (cells and fibers) (p<0.03 and p<0.02, respectively) and for VGF (p<0.003 for both parameters), when compared with non-diabetic controls (Figure 2, 10). For α-MSH, in contrast, no-significant changes were found. Both diabetics and non-diabetics showed a large variation in the number of positive cells (p  = 0.430589), as well as in the optical density (p  = 0.412148) (Figure 8, 10A).

As can be seen in figure 2 in the hypothalamic target areas of the arcuate nucleus also an increased staining for NPY can be observed reflecting the higher amount of peptide present in the fibers. In contrast to this increase in ARC staining is the observation in the NTS where a diminishment of NPY and VGF staining was observed. This result shows that the changes in NPY and VGF staining are not due to a general change in fixation or procedural artifact but is due to local peptide changes.

### NPY and VGF Show Lower Immunoreactivity in the NTS of Type-2 Diabetic Subjects

The NTS of the diabetic group shows a reduction in both the number of immuno reactive cells and as well as in the optical density of NPY (p<0.005 and p<0.001, respectively) and VGF (p<0.005 and p<0.001, respectively) (Figure3, 9, 10-B). Although there was a considerable variation of these two parameters in the diabetic group, the difference with the controls was highly significant. In contrast to NPY, TH showed a tendency to decrease in the NTS of diabetic individuals, but the difference with the controls did not reach significance (p  = 0.06 and p  = 0.51 for the difference in cell number and optical density, respectively). (Figure 4, 10-B).

### VGF co-localizes with NPY and α-MSH in the Human ARC

We examined the co-localization of VGF with both NPY and α-MSH in the human ARC and examined possible differences between controls and diabetics. VGF and NPY co-localize both in soma and fibers, especially in the medial (ventral and periventricular) zone of the nucleus (Figure 11). In the controls, VGF and α-MSH co-localize only sporadically in fibers and cell bodies within the ARC. However, in diabetics, (almost) all α-MSH neurons along the ventro lateral portion of the nucleus also contain VGF (Figure 12). VGF and α-MSH did not co-localize in the immuno reactive neurons clustered in the most ventro lateral part of the ARC, neither in controls nor in diabetics.

### NPY Co-localizes with VGF and TH in the Human Brainstem

The distribution of NPY, VGF and TH in the brainstem at the level of the dorso vagal complex was quite similar: neurons and fibers stained for these neuropeptides were found not only within the NTS and the surrounding area, but also along the trajectory of the Vagus nerve and the A1/C1 area (Figure 5). Because the TH cells showed a tendency to be diminished in diabetic brains we investigated whether there could be a difference in co-localization of TH with VGF or NPY. In the NTS, NPY co-localizes with VGF and TH (Figure 6 and 13). NPY was found to be present in the majority of the TH cell bodies in the A1 area (Figure 7). No differences were observed in the co-localization pattern of NPY, VGF and TH in diabetic brain as compared to the control brain.

## Discussion

### NPY Changes in the ARC, Related to Diabetes?

The increase in NPY immunoreactivity in the ARC of diabetic subjects is comparable to the increase of NPY under negative energy status as observed in rodent studies [5]–[8]. This suggests that in diabetic persons the NPY neurons react as if glucose levels are lower than normal. In rodents it has been shown that a variation in glucose and insulin signaling to NPY neurons of the ARC results in the generation of physiological responses that culminate in the regulation of hepatic glucose production and lipid metabolism via the autonomic system [8], [22], [26], [27]. Specifically, an increase in plasma glucose and insulin levels is followed by suppression of sympathetic activity to the liver resulting in lower glucose production [27]. Type-2 diabetes patients, instead, display inappropriately high hepatic glucose production, in spite of their hyperglycemic, hyperinsulinemic condition. This high hepatic glucose production is shown to be caused by increased sympathetic tone, which stimulates both glycogenolysis and gluconeogenesis [28], [29]. All in all, the present data suggest that the arcuate nucleus of diabetic patients perceives a negative metabolic state which may result in enhanced hepatic glucose production mediated by the sympathetic system.

### Technical Considerations

Animal experimental studies have indicated the arcuate nucleus as a major site for the control of metabolism and have suggested its importance for the development of diabetes. However one of the limitations of animal studies is the question whether these findings can be of relevance for human disease. The aim of the present study was to answer at least in part that question. However, also the present analysis of human brain material has several limitations that need to be taken into consideration for the interpretation of the data. First the conditions before and during death vary considerably and cannot be controlled as in animal experiments, in addition no information about body weight was available. Second, medication between the individuals, especially in the last phase of life may have varied enormously. Third, all measurements are done with immunohistochemical techniques and are therefore semi-quantitative and only reflect relative differences between the groups.

Yet, in spite of all these differences and putative problems, the diabetic group reacted quite distinct with respect to staining intensity and number of NPY and VGF neurons in the ARC as well as in the NTS. Moreover, this is not due to a general change in NPY of VGF immunoreactivity or change in neuron number, as can be concluded from the fact that in the NTS the opposite changes were noted as compared to the ARC. Self evidently more research will be needed to substantiate these findings but they indicate that indeed also in the human diabetic brain similar changes can be observed as in the diabetic rodent brain.

### Brain-liver Interplay

In rodents, inactivation of the insulin receptor, specifically in the periventricular area of the ARC causes an increase of NPY expression of about 50% and, at the same time, produces insulin resistance in the liver and increased hepatic glucose production, even in presence of high levels of insulin in plasma [30], [31].

According to the present data, the increase of NPY in the ARC in the human diabetic brain, suggests in addition to the peripheral insulin resistance as defined by the diabetic state, the presence hypothalamic insensivity to circulating insulin and/or glucose. At the other hand changes in leptin or other adipokines as occur in diabetes may also play a role in the observed changes in the ARC.

### NPY in the NTS

In the NTS of diabetic patients, NPY was decreased indicating a different mechanism of regulation between brainstem and hypothalamus. A comparable observation was made by Goncharuk *et al*, in 2007 [32] who reported that CRH neurons are activated in the hypothalamus of hypertensive patients, but not in the brainstem. The present observation suggests that in the NTS of diabetic subjects the proper inhibition of NPY neurons may take place by glucose and insulin suggesting that the gluco-sensing elements within the brainstem of diabetic subjects are functioning properly.

At the other hand our present results may indicate that the brainstem NPY neurons have a different role than those of the ARC. It seems logical to assume that both the NTS and ARC are able to sense and process the same information, i.e. glucose and insulin in the circulation, but will control glucose homeostasis using different mechanisms. Thus, the different NPY signaling as we observed in the NTS of diabetic patients might be the basis for a disruption in one of the multiple components of the homeostatic systems upstream of the NPY neurons.

### The POMC System

The contribution of POMC neurons in the control of glucose homeostasis has been a matter of controversy since a long time [33]–[37]. Thus the wide variation in α-MSH content observed in the ARC of both diabetic and control groups may be due to other factors, as mentioned in technical considerations above. These other effects may influence α-MSH synthesis, making it difficult to distinguish from the changes eventually triggered by hyperglycemia and/or hyperinsulinemia. At the other hand the present observation may suggest that in the human brain the POMC neurons may be less important for the inhibition of glucose production than in the rodent.

### VGF – Comparison with NPY and α-MSH

VGF, like NPY, is increased in the ARC of diabetic patients. This result agrees with the observation in mice that lack *vgf,* suggesting an anabolic role for VGF. VGF KO mice are lean and hyper metabolic, show increased energy consumption, and are resistant to several forms of obesity, hyperglycemia and hyperinsulinemia [10], [38]. Animal studies, suggest that both NPY and VGF are upregulated by negative metabolic state and are involved in sympathetic control of hepatic glucose production [10]. The present study also shows that the increase in VGF immunoreactivity coincides with an increase in NPY.

Remarkably α-MSH neurons in the ARC of diabetic patients increase their VGF content. Also in animals, VGF has been shown to be present in α-MSH neurons [39]. The increased co-localization of VGF and α-MSH indicate that, contrarily to what α-MSH immunoreactivity suggests, at least a portion of the POMC neurons is affected in the diabetic group.

### VGF in the NTS

For the first time, we report here not only the presence of VGF in the human brain but also that VGF and NPY are co-localized in a subset of neurons within the NTS. In addition, as in the ARC, both peptides follow a similar, though opposite pattern of immunoreactivity in the NTS.

The wide distribution of VGF in the brain, the large number of *vgf*-inducing factors, the complexity of the *vgf* ko phenotype and the co-expression of *vgf* in neuronal populations with opposite effects on metabolism do not allow a conclusion about the involvement of VGF in diabetes. However, we demonstrated that VGF, just as in rodents, is also present in the human brain in neurons that control energy balance and react to metabolic changes similar as NPY.

### Concluding Remarks

In the present study we demonstrated that type-2 diabetic patients, in spite of the hyperglycemia that characterizes this pathology, show an increased NPY content in the ARC, suggesting that the diabetic brain sensed a negative metabolic state. In view of the fact that many features of the NPY cells in the human ARC and NTS show similarities to the rodent NPY cells in these areas, we would like to propose that the changed perception of the metabolic condition in the ARC of diabetic persons might be the basis for these changes in NPY neurons. Hence, increased NPY signaling to second order neurons that control the activity of the sympathetic system may contribute to the anomalous, unbalanced hepatic glucose production.
